# L’Art Dentaire En Medecine Legale

**Published:** 1899-02

**Authors:** 


### Bibliography.

L'ART DENTAIRE EN MEDECINE LEGALE. By Dr. Oscar Amoëdo, Professor l'Ecole Odontotechnique de Paris. Masson et Cie, Paris, 1898.

The writer of this book states in his preface that he had for a long time been impressed with the close and important relations, legal and otherwise, existing between the professions of dentistry and medicine. After a careful study of the subject, and an exhaustive examination of the various works bearing upon it, he was impressed that writers upon medical jurisprudence had neglected to utilize the valuable contributions relating to it furnished by dental practitioners. With a view to remedy this, he had, during his sixteen years of continuous dental practice, collected many items of information from his own experience and the writings of others, intending at some time to embody them in a suitable presentation of this important question, when a disastrous catastrophy confirmed the importance of his undertaking.

The fire at the Bazar de la Charité, May 4, 1897, brought into prominence the important services rendered by members of the dental profession in identifying its unfortunate victims, and led him to at once make known his studies upon the subject in a communication to the last International Medical Congress at Moscow, and to the Dental Congress at Paris. The importance of the subject demanded more than this. He therefore made a further study of the subject, a more thorough research, and a more extended use of the material at command. The information thus gained is embodied in the work in question.

He first treats upon the nomenclature of dental anatomy, following closely the suggestion of Dr. Black in his report on this subject to the Columbian Dental Congress. This is followed by dental anatomy, general and descriptive; dental anomalies; facial peculiarities of the human teeth, and peculiarities of the jaws and teeth of idiots, prostitutes, criminals, dwarfs, and other degenerates.

He then considers the various conditions, normal or abnormal, general or local, having a more or less direct bearing upon the teeth, and the various disorders caused or aggravated by the position or condition of the dental organs. Dental caries, erosion, the effects of various occupations upon the teeth; fractures and luxations of the teeth, and the medico-legal questions which may arise therefrom; bites or wounds made with the teeth in general, those made by man or by animals, and the liability of thereby transmitting diseases; the wear of the teeth, and the teeth after death, are each in turn duly considered, not only as these several conditions are presented to and concern the dentist, but also as they may and do at times assume a legal importance.

Under the heading, "The Dentist as an Expert," he treats of dental jurisprudence in five sections,—1, the dentist as an expert; 2, accidents due to extraction; 3, anæsthetics, general and local, in dental surgery; 4, infections communicated by the dentist; 5, identification of the dead by the expert dentist.

He devotes considerable space to the dental evidence submitted in identifying the unfortunate victims of the charity bazar, to dental notation, and the care to be observed in recording dental operations, so that when called upon to identify the living or the dead the dentist may be able to give unimpeachable testimony.

The volume closes with a very full and instructive bibliography of each subject considered. As a help to future students this will prove useful and instructive. In a few instances, however, the names are misspelled, or the initials in part omitted.

In treating each subject the author has endeavored to give the dento-medical, or dento-legal, rather than the purely legal or purely dental aspect, and this constitutes the real value of the book. In this it is unique. The author well merits the thanks of the profession for his patient and thorough research. He has not only given them a useful book, but has made the path easier for those who may follow the same line of study.

